# Molluscs for Sale: Assessment of Freshwater Gastropods and Bivalves in the Ornamental Pet Trade

**DOI:** 10.1371/journal.pone.0161130

**Published:** 2016-08-15

**Authors:** Ting Hui Ng, Siong Kiat Tan, Wing Hing Wong, Rudolf Meier, Sow-Yan Chan, Heok Hui Tan, Darren C. J. Yeo

**Affiliations:** 1 Department of Biological Sciences, National University of Singapore, Singapore; 2 Lee Kong Chian Natural History Museum, National University of Singapore, Singapore; 3 The Division of Biology and Biomedical Sciences, Washington University in St. Louis, United States of America; 4 VBox 888313, Singapore; University of Arkansas Fayetteville, UNITED STATES

## Abstract

The ornamental pet trade is often considered a key culprit for conservation problems such as the introduction of invasive species (including infectious diseases) and overharvesting of rare species. Here, we present the first assessment of the biodiversity of freshwater molluscs in the ornamental pet trade in Singapore, one of the most important global hubs of the ornamental aquarium trade, and discuss associated conservation concerns. We recorded freshwater molluscs from ornamental pet shops and major exporters including non-ornamental species (e.g., hitchhikers, molluscs sold as fish feed). We recorded an unexpectedly high diversity—59 species—of freshwater bivalves and gastropods, with the majority (38 species or 64%) being from the Oriental region. In addition to morphological examination, we sequenced the DNA barcode region of mitochondrial CO1 and 16S genes to provide molecular data for the confirmation of the identification and for future re-identification. DNA barcodes were obtained for 50 species, and all but four were separated by > 3% uncorrected pairwise distances. The trade has been considered a main introduction pathway for non-native species to Singapore, and we found that out of 15 species in the trade as well as in the wild in Singapore, 12 are either introduced or of unknown origin, representing almost half of the known non-native freshwater molluscs in Singapore. Particularly prevalent are non-ornamental species: six hitchhikers on aquarium plants and six species sold as fish feed. We found that a quarter of the trade species have a history of introduction, which includes 11 known or potentially invasive species. We conclude that potential overharvesting is difficult to assess because only half of the trade species have been treated by IUCN. Of these, 21 species are of Least Concern and three are Data Deficient. Our checklist, with accompanying DNA barcodes, images, and museum vouchers, provides an important reference library for future monitoring, and constitutes a step toward creating a more sustainable ornamental pet trade.

## Introduction

The global aquarium or ornamental fish industry has been valued at US$15 billion per year [1]. While fish in the ornamental pet trade have generally been well catalogued and studied, (e.g., [2, 3, 4], little information exists for invertebrates, including freshwater molluscs, which have seen noticeably increased interest from hobbyists in recent years [5, 6, 7]. Few accounts of non-fish taxa in the trade have been published, and most of these concentrate on the ornamental trade as a source or pathway of introduction of organisms (e.g., [8–15]). Invasive species are often noted in the ornamental pet trade only after they have established populations, or have caused negative impacts in a new range (e.g., [8, 16]). The few freshwater molluscs reported from the trade have mainly been species identified as intermediate hosts of zoonotic parasites [8, 16, 17, 18, 19]. Despite the risks related to the spread of freshwater molluscs via the pet trade, ornamental freshwater molluscs continue to escape in-depth scrutiny.

In addition to being implicated in the introduction of non-native species, the ornamental pet trade has also been associated with overharvesting of species, especially of fish [20, 21, 22]. Overexploitation of freshwater molluscs is a neglected, but genuine issue, because many species have highly-restricted distributions, strict habitat requirements, or both [23, 24, 25]. Non-marine molluscs are recognised as being highly threatened—99% of documented mollusc extinctions are of non-marine species, yet fewer than 2% of mollusc species have been properly assessed [26]. This makes it likely that the correct number of extinctions is much higher than estimated [27]. Demand for narrowly-endemic species in the ornamental pet trade has increased recently [28] and may represent a conservation concern that will remain difficult to detect until there is a concerted effort to document the freshwater molluscs in the trade.

While freshwater molluscs in the ornamental pet trade have largely been overlooked, countries have been implementing measures to monitor other commonly-traded animals for 1) biosecurity to prevent the introduction of potentially harmful organisms, including vectors of infectious diseases, or 2) law enforcement to protect against illegal wildlife trade [29–32]. One of the main challenges in monitoring organisms that are imported or exported is the difficulty in reliably identifying those species that are of biosecurity or conservation concern. To address this problem, molecular tools such as DNA barcoding are increasingly being used for rapid and accurate identification of ornamental organisms, especially the employment of DNA barcoding for fish [33, 34]. The mitochondrial cytochrome oxidase I (CO1) and 16S rRNA genes have been used for this purpose and for tracing source populations of species in the ornamental trade [33–39].

Biosecurity measures are of primary importance to countries involved in the ornamental pet industry. Singapore is the top exporter of ornamental fish in the world, with annual trade of ornamental freshwater fish alone averaging over US$60 million [40, 41]. The United States of America and the United Kingdom receive 40% of Singapore’s exports, and among the top 20 trading partners are countries from across Asia, Europe, Africa, and Australasia [41]. Biosecurity efforts are usually focusing on preventing entry and the spread of infectious diseases [31, 42]. A related concern is that shipments of ornamental fish or plants could also carry hitchhiking organisms. Snails are regularly intercepted by border control worldwide, including in shipments originating from Singapore [19, 43, 44]. Although the ornamental pet trade has been suspected to be the main source of the many non-native freshwater molluscs established in Singapore [45–48], the link remains speculative [47].

We present here the first assessment of freshwater mollusc species acquired from the ornamental pet trade. We included both intentionally imported ornamental species as well as species that are accidentally brought via the trade. As Singapore is a global hub for the ornamental pet trade, the resulting species list is potentially representative of species that could be traded and potentially introduced worldwide. Additionally, we provide the first set of CO1 and 16S DNA barcodes of freshwater molluscs present in the ornamental trade for a nascent DNA reference library of freshwater molluscs in the ornamental pet trade. Such a library could provide critical information for the conservation of freshwater molluscs (e.g., to monitor if any threatened species are being traded [38]), and to aid in biosecurity monitoring and prevention of the introduction of harmful alien species [34].

## Material and Methods

### Sample and data collection

We recorded freshwater mollusc species encountered in the Singapore ornamental pet trade—local ornamental pet retail shops and major ornamental exporters (see S1 Table)—between 2008 and 2014. “Ornamental exporters” refers to major distributors that import stock from various sources (especially from within the surrounding region), and re-package the animals for export to customers abroad [49]. Species were considered as hitchhiking species when they were found in shipments or tanks that did not specifically contain those molluscs for sale. This includes hitchhikers from tanks holding other mollusc or fish species, and hitchhikers on ornamental aquatic plants in home aquariums or nurseries. Specimens that were being sold primarily as fish feed were also included in the study. Voucher specimens are deposited in the Zoological Reference Collection (ZRC) of the Lee Kong Chian Natural History Museum at the National University of Singapore (ZRC.MOL.5904–5952, 6284–6333, 6752–6754; see Results). At least two individuals per lot (except for hitchhikers, in which case there were mostly one individual per lot) were examined, and identified based on descriptions and figures in [47, 48, 50–75], and checked against original descriptions of the taxa. The distribution, introduction history, and associated parasites of all species were recorded based on available literature (see Results). When necessary, we consulted malacological experts of respective taxa, for advice and assistance with identification.

### DNA extraction, amplification, and sequencing

We extracted total genomic DNA from the tissue samples (foot tissue of gastropods or adductor muscle of bivalves) of freshwater molluscs obtained from the aquarium trade using a phenol-chloroform extraction protocol. The DNA barcodes (mitochondrial CO1 and 16S rRNA) were amplified in polymerase chain reactions (PCR) with a total volume of 23–24μl PCR rection mixture (2.5μl of Taq 10× buffer, 2mM dNTPs, 1μl of 10μM primers (Table 1), 0.25μl of BioReady rTaq DNA Polymerase (Bulldog Bio), and DNase-free sterile water), at 95°C for 5min, 34 cycles of 95°C for 30sec, 45–48°C for 30sec, and 72°C for 30sec, and a final extension of 72°C for 10min. Fragments of CO1 were obtained using three different pairs of primers that enabled us to assess the wide range of taxa involved (Table 1). The primer pairs LCO1490/HCO2198 and GASF1_t1/GASR1_t1 amplified approximately 600 base pairs (bp) in the barcode region, while mlCOintF/jgHCO2198 amplified a shorter fragment (313 bp) in the 3’ region. The latter pair was used in specimens that failed to amplify the standard barcode region. The size of 16S fragments amplified ranged from 320 to 476 bp. The PCR products were checked visually on a 1% agarose gel. Post PCR clean-ups were performed on successfully amplified products using SureClean reagent (Bioline Inc.) following the manufacturer’s recommendations. The purified products were sequenced with BigDye Terminator reactions and analysed on the ABI PRISM 3130XL sequencer (Applied Biosystems) at the DNA Sequencing Laboratory of the National University of Singapore.

**Table 1:** Primers used to obtain sequences.

Primers	Sequences	References
LCO1490	5′ GGTCAACAAATCATAAAGATATTGG 3′	[120]
HCO2198	5′ TAAACTTCAGGGTGACCAAAAAATCA 3′	[120]
GASF1_t1	5′ TGTAAAACGACGGCCAGTTTTCAACAAACCATAARGATATTGG 3′	[121]
GAS R1_t1	5′ CAGGAAACAGCTATGACACTTCWGGRTGHCCRAARAATCARAA 3′	[121]
jgHCO2198	5’ TAIACYTCIGGRTGICCRAARAAYCA 3’	[122]
mICOintF	5’ GGWACWGGWTGAACWGTWTAYCCYCC 3’	[123]
16Sar-L	5’ CGCCTGTTTATCAAAAACAT 3’	[124]
16Sbr-H	5’ CCGGTCTGAACTCAGATCACGT 3’	[124]

### Data analysis

We visually inspected and trimmed sequences using Sequencher ver. 4.6 (Genecodes). The CO1 and 16S genes were aligned using MAFFT version 7 [76] with default settings. Aligned CO1 sequences were checked for translatability into amino acids and were gap free. DNA sequences were inspected using objective clustering in SpeciesIdentifier version 1.7.9 [77], with species delimitation thresholds of 1–4% uncorrected pairwise distances [78, 79]. Objective clusters are groups of sequences which have at least one other sequence below the threshold. Each cluster is considered as a molecular operational taxonomic unit (mOTU) [77, 80]. Previous studies [78, 81] have shown the intraspecific genetic distance for majority of gastropods is <2% for CO1. Here, we employed 1–4% thresholds to assess for stability of genetic clusters. A BLASTn search (highly similar sequences (megablast)) [82] was carried out on GenBank in order to confirm the species delimited in SpeciesIdentifier. If identified sequences from GenBank were 97–100% identical to the query sequence, but the species identity differed from our morphology-based identification, we re-examined the specimens and compared it to the original descriptions. Wherever possible, we contacted the contributors of the GenBank sequences for comparative material (photographs or material deposited in collections). All sequences were deposited in GenBank and BOLD (S2 Table).

## Results

### Source of freshwater molluscs in the trade

**Fig 1:**
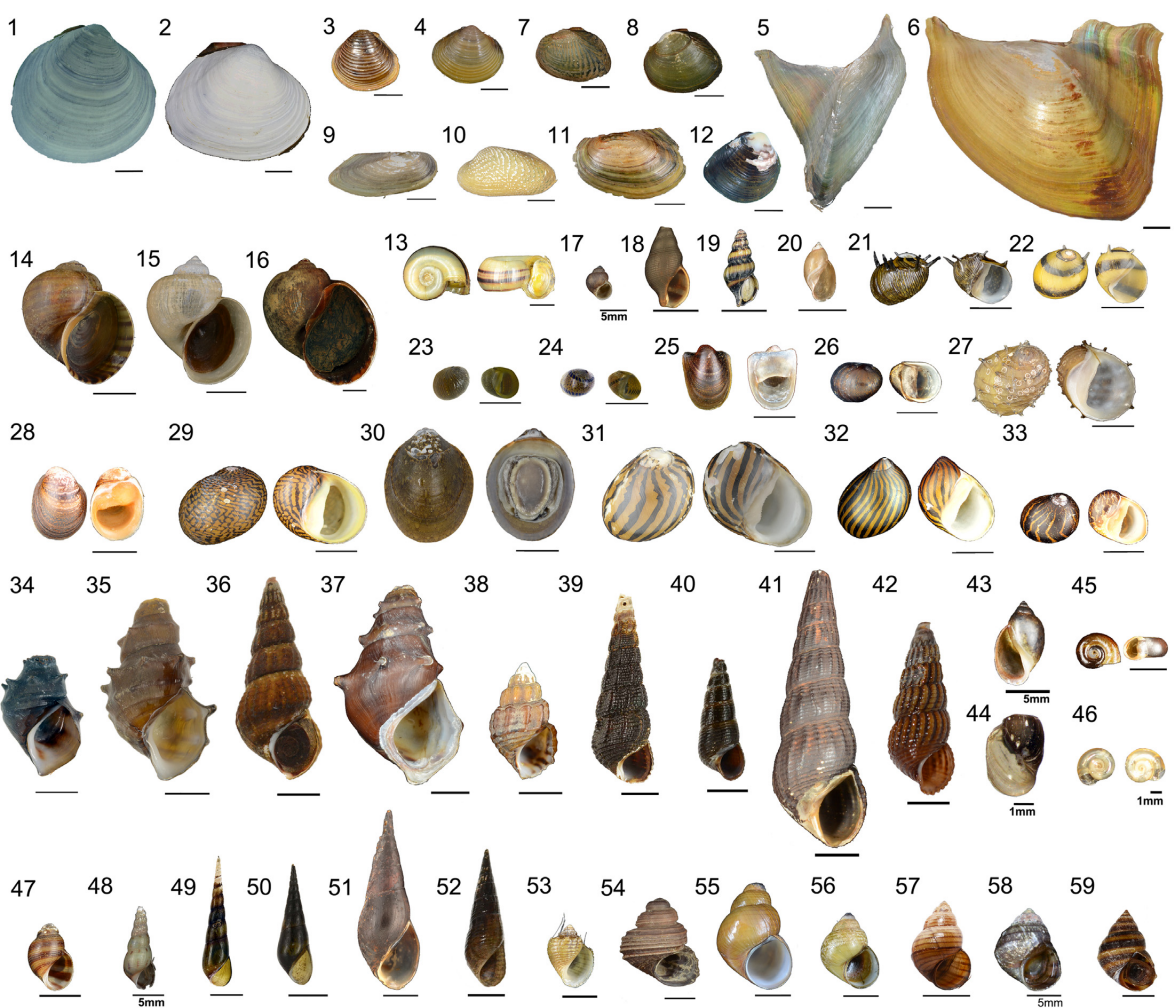
Freshwater molluscs in the ornamental pet trade. Unless indicated differently, scale bars = 10mm. 1. *Batissa similis*; 2. *Batissa violacea*; 3. *Corbicula fluminea*; 4. *Corbicula moltkiana*; 5. *Hyriopsis bialata*; 6. *Hyriopsis desowitzi*; 7. *Parreysia burmana*; 8. *Parreysia tavoyensis*; 9. *Pilsbryoconcha exilis*; 10. *Scabies crispata*; 11. *Sinanodonta woodiana*; 12. *Unionetta fabagina*; 13. *Marisa cornuarietis*; 14. *Pomacea canaliculata*; 15. *Pomacea diffusa*; 16. *Pomacea maculata* (photograph by K.A. Hayes); 17. *Bithynia sp*.; 18. *Clea bockii*; 19. *Clea helena*; 20. *Radix rubiginosa*; 21. *Clithon corona*; 22. *Clithon diadema*; 23. *Clithon lentiginosum*; 24. *Clithon mertoniana*; 25. *Neripteron auriculata;* 26. *Neritina iris*; 27. *Neritina juttingae*; 28. *Neritina violacea*; 29. *Neritodryas cornea*; 30. *Septaria porcellana*; 31. *Vittina coromandeliana*; 32. *Vittina turrita*; 33. *Vittina waigiensis*; 34. *Brotia armata*; 35. *Brotia binodosa*; 36. *Brotia herculea*; 37. *Brotia pagodula*; 38. *Sulcospira tonkiniana*; 39. *Tylomelania towutica*; 40. *Tylomelania* sp.; 41. *Tylomelania* sp.; 42. *Tylomelania* sp.; 43. *Physa* sp.; 44. *Amerianna carinata*; 45. *Indoplanorbis exustus*; 46. *Gyraulus convexiusculus*; 47. *Semisulcospira* sp.; 48. *Melanoides tuberculata*; 49. *Stenomelania offachiensis*; 50. *Stenomelania plicaria*; 51. *Stenomelania cf*. *plicaria*; 52. *Stenomelania* sp.; 53. *Thiara cancellata*; 54. *Celetaia persculpta;* 55. *Filopaludina cambodjensis*; 56. *Filopaludina peninsularis*; 57. *Filopaludina polygramma*; 58. *Sinotaia guangdungensis*; 59. *Taia pseudoshanensis*.

**Fig 2:**
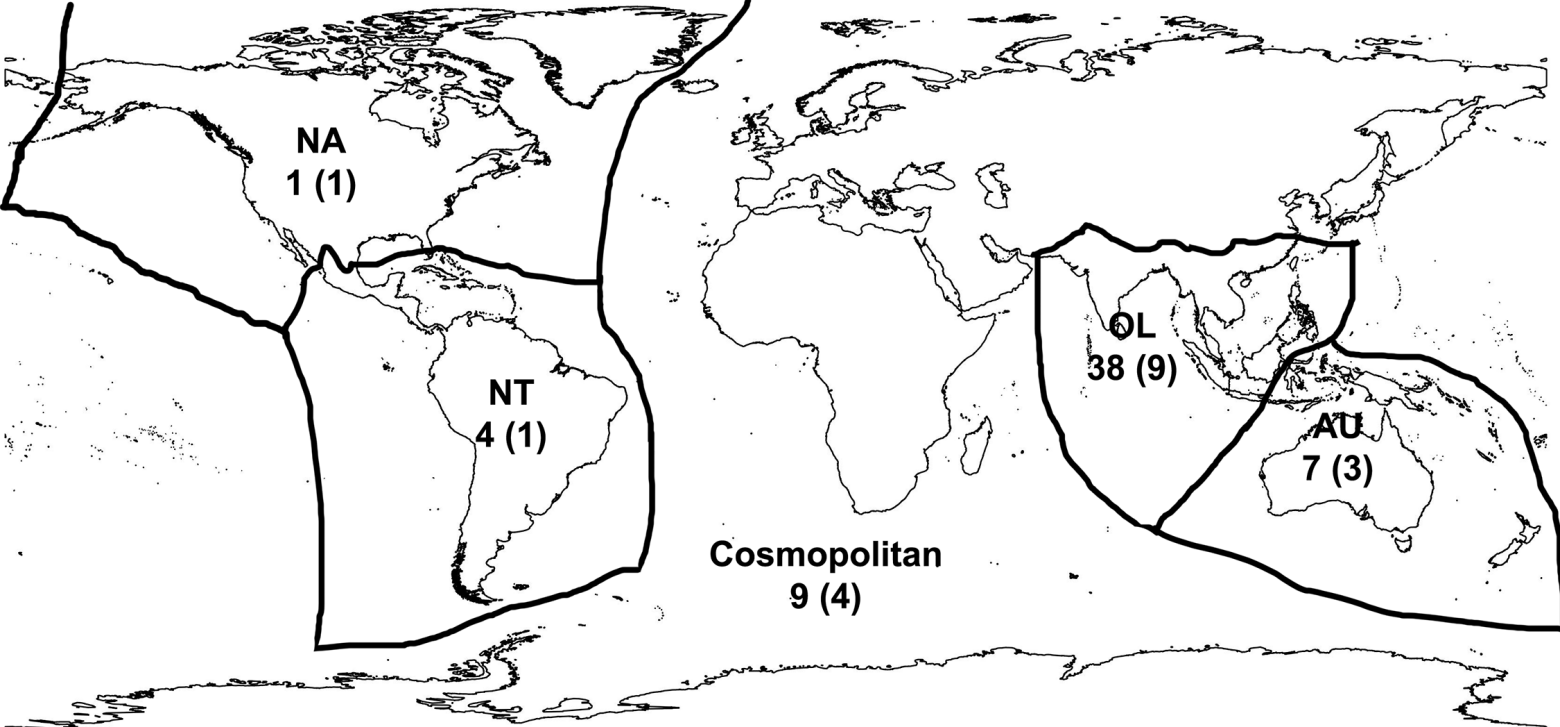
Native distribution of freshwater mollusc species in the ornamental pet trade. Numbers indicate the number of species from each zoogeographic region; numbers in brackets are the number of families. Zoogeographic regions follow [24].

We obtained 148 lots of freshwater molluscs, and identified 59 species from 13 families in the ornamental pet trade based on morphology (Table 2, Fig 1). Fifty species in the trade originate from one of four zoogeographic regions (Fig 2)—38 species from the Oriental region (including Sundaic Southeast Asia, Indo-Burma, and the Indian subcontinent), seven from the Australasian region (including Sulawesi), four from the Neotropics, and one from the Nearctic—while nine species are regarded as being native to more than one region (cosmopolitan). Fourteen species from seven families have been introduced to regions beyond their native distributions, with 11 of these recorded to cause (or potentially cause) negative impacts in their invaded habitats (Table 3). Twenty-four species have been assessed for conservation status by the IUCN, with 21 being species of Least Concern (LC), and three species being Data Deficient (DD) [83].

**Table 2:** Checklist of freshwater molluscs recorded from the Singapore ornamental pet trade from 2008 to 2014.

Family	Species	Geographic Distribution	Source		IUCN Status	DNA barcode	Catalogue No.	Remarks	References
			E	L					
Cyrenidae	*Batissa similis* Prime, 1862	OL	1		DD		ZRC.MOL.6752		[58]
	*Batissa violacea* (Lamarck, 1818)	OL	1			CO1; 16S	ZRC.MOL.5904		[51]
	*Corbicula fluminea* (Müller, 1774)†	OL	1	1	LC	CO1; 16S	ZRC.MOL.5905	Hitchhiker and feed.	[56]
	*Corbicula moltkiana* Prime, 1878	OL		1	DD	CO1; 16S	ZRC.MOL.5907		[61]
Unionidae	*Hyriopsis bialata* (Simpson, 1800)	OL	1	1	LC	CO1	ZRC.MOL.5908		[55]
	*Hyriopsis desowitzi* Brandt, 1974	OL	1		DD	16S	ZRC.MOL.2195		[55]
	*Parreysia burmana* (Blandford, 1869)	OL	1		LC	CO1; 16S	ZRC.MOL.5909		[55, 58]
	*Parreysia tavoyensis* (Gould, 1843)	OL	1			CO1; 16S	ZRC.MOL.5910		[58]
	*Pilsbryoconcha exilis* (Lea, 1856)†	OL	1		LC	CO1	ZRC.MOL.5911		[47, 55, 125]
	*Scabies crispata* (Gould, 1843)	OL	1		LC	16S	ZRC.MOL.5913		[55, 58]
	*Sinanodonta woodiana* (Lea, 1834)†	OL	1		LC	CO1	ZRC.MOL.2914		[47, 56, 126, 127]
	*Unionetta fabagina* (Deshayes & Jullien, 1874)	OL	1		LC		ZRC.MOL.67523		[55, 128]
Ampullariidae	*Marisa cornuarietis* (Linnaeus, 1758)	NT		1	LC	CO1	ZRC.MOL.5914		[66, 129, 130]
	*Pomacea canaliculata* (Lamarck, 1822)†	NT	1	1	LC	CO1	ZRC.MOL.5915	Feed.	[67, 130]
	*Pomacea maculata* Perry, 1810†	NT		1			Collection of K.A. Hayes		[48, 66] (as *Pomacea insularum*), [67] (as *Pomacea insularum*), [72]
	*Pomacea diffusa* Blume, 1957	NT	1	1		CO1	ZRC.MOL.5916		[66, 67, 129, 130]
Bithynidae	*Bithynia* sp.†	OL, PA, AU, AT		1		CO1; 16S	ZRC.MOL.5917	Feed.	[47]
Lymnaeidae	*Radix rubiginosa* (Michelin, 1831)	OL		1		CO1; 16S	ZRC.MOL.5920		[55]
Nassariidae	*Anentome bockii* (Brot, 1881)	OL	1			CO1	ZRC.MOL.5918		[131]
	*Anenetome helena* (Philippi, 1847)	OL	1			CO1	ZRC.MOL.5919		[55]
Neritidae	*Clithon corona* (Linnaeus, 1758)	OL	1	1	LC	CO1	ZRC.MOL.5921		[53, 75]
	*Clithon diadema* (Récluz, 1841)	OL	1			CO1; 16S	ZRC.MOL.5922		[58, 75]
	*Clithon lentiginosum* (Reeve, 1855)	OL, AU	1			CO1; 16S	ZRC.MOL.5923		[75]
	*Clithon mertoniana* (Récluz, 1843)	OL	1			CO1; 16S	ZRC.MOL.5924		[75]
	*Neripteron auriculata* (Lamarck, 1816)	OL, AU	1			CO1; 16S	ZRC.MOL.5925		[53, 75]
	*Neritina iris* (Mousson, 1849)	OL, AU	1			CO1	ZRC.MOL.5926		T. Eichhorst, pers. comm.
	*Neritina juttingae* Mienis, 1973	OL	1			CO1; 16S	ZRC.MOL.5927		[132]
	*Neritina violacea* (Gmelin, 1791)† ^B^	OL, AU, PA	1		LC	CO1	ZRC.MOL.5928		[53]
	*Neritodryas cornea* (Linnaeus, 1758)	OL to PAC	1			CO1	ZRC.MOL.5929		[55]
	*Septaria porcellana* (Linnaeus, 1758)	OL		1		16S	ZRC.MOL.5933		[110]
	*Vittina coromandeliana* (Sowerby, 1932)† ^B^	OL	1			CO1	ZRC.MOL.5930		[55]
	*Vittina turrita* (Gmelin, 1791)	OL to PAC	1			CO1	ZRC.MOL.5931		[53]
	*Vittina waigiensis* (Lesson, 1831)	OL	1			CO1; 16S	ZRC.MOL.5932		[53]
Pachychilidae	*Brotia armata* (Brandt, 1968)	OL	1		LC	16S	ZRC.MOL.2935	Narrowly-endemic	[64]
	*Brotia binodosa* (Blanford, 1903)	OL	1		LC	16S	ZRC.MOL.2934	Narrowly-endemic	[55]
	*Brotia herculea* (Gould, 1846)	OL	1		LC	CO1	ZRC.MOL.5934		[64]
	*Brotia pagodula* (Gould, 1847)	OL	1				ZRC.MOL.5935	Narrowly-endemic	[55]
	*Sulcospira tonkiniana* (Morlet, 1887)	OL	1		LC	CO1	ZRC.MOL.5936		[70]
	*Tylomelania towutica* (Kruimel, 1913)	AU	1			CO1	ZRC.MOL.5939	Narrowly-endemic	[133]
	*Tylomelania* sp.	AU	1			CO1, 16S	ZRC.MOL.2397	Narrowly-endemic	T. von Rintelen, pers. comm.
	*Tylomelania* sp.	AU	1				ZRC.MOL.5939	Narrowly-endemic	T. von Rintelen, pers. comm.
	*Tylomelania* sp.	AU	1				ZRC.MOL.2933	Narrowly-endemic	T. von Rintelen, pers. comm.
Physidae	*Physa* sp.	NA	1	1		CO1	ZRC.MOL.5773	Hitchhiker.	[74, 134]
Planorbidae	*Amerianna carinata* (H. Adams, 1861)†	AU		1			ZRC.MOL.5952	Feed and hitchhiker.	[47, 53, 55, 135, 136]
	*Gyraulus convexiusculus* (Hutton, 1849)	OL, PA		1	LC	CO1	ZRC.MOL.5953	Hitchhiker.	[137]
	*Indoplanorbis exustus* (Deshayes, 1834)†	OL		1	LC	CO1	ZRC.MOL.5940	Feed.	[53, 57]
Semisulcospiridae	*Semisulcospira* sp.	OL	1			CO1	ZRC.MOL.5942		[56], F. Köhler pers. comm.
Thiaridae	*Melanoides tuberculata* (Müller, 1774)†	OL, AU, PA		1	LC	CO1	ZRC.MOL.5943		[19, 55, 138]
	*Stenomelania offachiensis* Lesson, 1831	AU	1			CO1; 16S	ZRC.MOL.5944		[112]
	*Stenomelania plicaria* (Born, 1778)	OL	1		LC	CO1	ZRC.MOL.5945		[112]
	*Stenomelania* cf. *plicaria*	OL	1			CO1	ZRC.MOL. 2195		[112]
	*Stenomelania* sp.	OL, AU, PA, PAC	1			CO1; 16S	ZRC.MOL. 5946		M. Glaubrecht, pers. comm.
	*Thiara cancellata* Röding, 1798	OL	1			CO1; 16S	ZRC.MOL.5947		[53]
Viviparidae	*Celetaia persculpta* (Sarasin & Sarasin, 1898)	AU		1			ZRC.MOL.6752	Narrowly-endemic	[139]
	*Filopaludina cambodjensis* (Mabile & Le Mesle, 1866)	OL		1	LC	CO1	ZRC.MOL.5948	Hitchhiker.	[55]
	*Filopaludina peninsularis* Brandt, 1974†	OL		1	LC	CO1; 16S	ZRC.MOL.5949	Hitchhiker.	[47, 55]
	*Filopaludina polygramma* (Martens, 1897)†	OL		1			ZRC.MOL.2721		[47, 55]
	*Sinotaia guangdungensis* (von Frauenfeld, 1862)†	OL		1		CO1	ZRC.MOL.5950	Feed.	[47] (as *Taia polyzonata*), [73, 140] (as *Bellamya heudei guangdungensis*)
	*Taia pseudoshanensis* Zilch, 1955	OL	1			16S	ZRC.MOL.5951		[52]

† indicates species found in Singapore

^B^ indicates brackish water species. Geographic distribution: NA—Nearctic; NT—Neotropical; PA—Palaearctic; AT—Afrotropical; OL—Oriental; AU—Australasian; PAC—Pacific Oceanic Islands. Source (of specimens): E—export; obtained from ornamental exporters; L—local; obtained from local pet shops or aquatic plant nurseries. IUCN status (if assessed): LC—Least Concern; DD—Data Deficient. DNA barcode: CO1—mitochondrial cytochrome oxidase subunit 1 gene; 16S—16S ribosomal RNA gene; refer to S2 Table for further details. Catalogue no.: ZRC—Zoological Reference Collection of the Lee Kong Chian Natural History Museum. Remarks include: hitchhiker—hitchhikers among other ornamental animals or plants; feed—sold as fish food; narrowly-endemic—species that are endemic to a particular river system or lake.

**Table 3:** Freshwater molluscs in the aquarium trade with history of introduction.

Family	Species	Native to	Introduced to	Known or potential impacts	References
Cyrenidae	Corbicula fluminea*	Unknown	Worldwide	Outcompete native species; biofouling	[141–146]
Unionidae	Pilsbryoconcha exilis*	OL	Singapore and Philippines	—	[47, 125]
	*Sinanodonta woodiana*	OL	Europe, Indonesia, Singapore, Central America, USA	Displaced native species; compete with native species for food, habitats, fish hosts	[47, 126, 128, 147–150]
Ampullariidae	Marisa cornuarietis*	NT	USA, Caribbean islands, Central America, Africa, Middle East, Spain	Agricultural pest	[66, 129, 130, 151]
	Pomacea canaliculata*	NT	Worldwide	Agricultural pest; outcompete native species; co-introduction of zoonotic parasite *Angistrongyliasis cantonensis* resulted in outbreaks of angiostrongyliasis when snail consumed by humans	[67, 72, 130, 152–155]
	Pomacea diffusa*	NT	USA, Brazil, Colombia, Panama, French Guiana, Israel, South Africa, Australia, New Zealand, India, Sri Lanka	Potential predator and competitor of native species; known to feed on aquatic macrophytes, snail eggs.	[44, 66, 67, 129] (as *Pomacea bridgesii*), [130, 156] (as *Pomacea bridgesii*)
	Pomacea maculata*	NT	Southeast Asia, South Korea, USA	Agricultural pest; outcompete native species; co-introduction of zoonotic parasite *Angistrongyliasis cantonensis*	[67] (as *Pomacea insularum*), [72, 130] (as *Pomacea insularum*), [157] (as *Pomacea insularum*), [158, 159
Lymnaeidae	Radix rubiginosa*	OL	UK, Israel, South Africa	Known host of various parasites that affect humans and livestock	44, 160–162]
Planorbidae	Amerianna carinata*	AU	India, Java, Martinique island, Nigeria, Singapore, Thailand, Japan	—	[47, 135, 163]
	Gyraulus convexiusculus*	OL	Japan, Middle East	Known host of zoonotic parasites Echinostomatidae	[71, 137]
	Indoplanorbis exustus*	OL	Nigeria, Java and Sulawesi, of unknown origin in Singapore	Known host of zoonotic parasite *Schistosoma spindale*	[47, 50, 53, 57, 164]
Thiaridae	Melanoides tuberculata*	OL, AU, PA	Worldwide	Outcompeted native species; co-introduction of various parasites including zoonotic parasites and *Centrocestus formosanus* that has resulted in losses in commercial fisheries and infected wild fish populations	[19, 89, 138, 165, 166]
Viviparidae	Filopaludina polygramma*	OL	Singapore	Known host of zoonotic parasite *Angiostrongyliasis cantonensis*	[47, 71]
	*Sinotaia guangdungensis*	OL	Australia, Singapore, Malaysia	—	[47] (as *Taia polyzonata*), [73, 140] (as *Bellamya heudei guangdungensis*)

*Purportedly introduced via the ornamental pet trade.

Twenty-one of the species were recorded from aquarium shops or ornamental plant nurseries in Singapore, while 45 were recorded from ornamental exporters. Of these, five species were recorded from both sources. Six species were found as hitchhikers on aquatic plants or incidentally transported with ornamental fish or other freshwater molluscs (“hitchhiker”). Fifteen of the species are found in the wild in Singapore—three are native species (*Melanoides tuberculata*, *Neritina violacea*, *Vittina coromandeliana*), eight introduced (*Amerianna carinata*, *Corbicula fluminea*, *Filopaludina polygramma*, *Pilsbryoconcha exilis*, *Pomacea canaliculata*, *Pomacea maculata*, *Sinanodonta woodiana*, *Sinotaia guangdungensis*), and four species are of unknown origin (*Bithynia* sp., *Indoplanorbis exustus*, *Filopaludina peninsularis*, *Radix rubiginosa*). Two of the native species, *Neritina violacea* and *Vittina coromandeliana*, are only found in brackish water habitats in Singapore [69], and are excluded from further discussion. Among the introduced species that are established in Singapore, seven are sold as fish feed (*Amerianna carinata*, *Bithynia* sp., *Corbicula fluminea*, *Indoplanorbis exustus*, *Melanoides tuberculata*, *Pomacea canaliculata*, *Sinotaia guangdungensis*). They are sold cheaply in mixed-species bags (<US$1/bag), and appear to have been collected from locally-established populations (i.e., not imported).

### DNA barcodes

DNA barcodes were successfully amplified for 50 of the 59 recorded species (17 species have both CO1 and 16S sequences, 27 only CO1, and seven only 16S) (Table 4, S2 Table). Fresh samples could not be obtained for some species, and they could thus not be sequenced. Overall, there is high congruency between the species identity based on morphology, and mOTU (Table 4). For CO1, all 44 morphologically-identified species were congruent with molecular data at 1–3% thresholds (Table 4). Within the same threshold, the 24 16S morphological species were grouped into 22 clusters (i.e., mOTU) (Table 4). Most of the mOTUs remained stable for CO1 until up to 8–9% thresholds, except for *Clithon corona* and *Clithon lentiginosum* that were separated by 3.5% uncorrected pairwise distance. Morphology and genetic data were incongruent for two 16S mOTUs, with two morphological species each being lumped into one genetic cluster, even at 1% threshold: *Brotia armata* and *Brotia binodosa* were separated by 0.5% uncorrected pairwise distance, while *Corbicula fluminea* and *Corbicula moltkiana* were separated by 0.8%.

**Table 4:** Comparison of morphologically-identified species with sequence clusters (mOTU) for CO1 and 16S at 1–4% thresholds, and corresponding top hits on BLAST above 96% identity.

Identity Based on Morphology		CO1		16S	
Family	Species	Congruence with sequence clusters	Top Hits on BLAST (> 96% identity)	Congruence with sequence clusters	Top Hits on BLAST (> 96% identity)
Cyrenidae	*Batissa violacea*	Yes (1–4%)	*Batissa violacea* DQ837727 (97)	Yes (1–4%)	—
	*Corbicula* cf. *fluminea*	Yes (1–4%)	*Corbicula sp*. GU781082 (100)	No (1–4%)	*Corbicula colorata* JX399588 (99)
	*Corbicula* sp.	Yes (1–4%)	*Corbicula moltkiana* AY275660.1 (100)	No (1–4%)	*Corbicula largillierti* AB522658 (99)
Unionidae	*Hyriopsis bialata*	Yes (1–4%)	—	N/A	N/A
	*Hyriopsis desowitzi*	N/A	N/A	Yes (1–4%)	—
	*Parreysia burmana*	Yes (1–4%)	—	Yes (1–3%), no (4%)	*Parreysia olivacea* KP795044 (96)
	*Parreysia* sp.	Yes (1–4%)	*Parreysia tavoyensis* JN243901 (99)	Yes (1–3%), no (4%)	*Parreysia tavoyensis* KP795043 (99)
	*Pilsbryoconcha exilis*	Yes (1–4%)	— (matched to same species KP795024 at 90%)	N/A	N/A
	*Scabies crispata*	N/A	N/A	Yes (1–4%)	*Scabies crispata* KP795048 (97)
	*Sinanodonta woodiana*	Yes (1–4%)	*Sinanodonta woodiana* KJ434487 (99)	N/A	N/A
Ampullariidae	*Marisa cornuarietis*	Yes (1–4%)	*Marisa cornuarietis* KM100140 (98)	N/A	N/A
	*Pomacea canaliculata*	Yes (1–4%)	*Pomacea canaliculata* KJ739609 (99)	N/A	N/A
	*Pomacea diffusa*	Yes (1–4%)	*Pomacea diffusa* EF515065 (100)	N/A	N/A
Bithyniidae	*Bithynia* sp.	Yes (1–4%)	—	Yes (1–4%)	—
Lymnaeidae	*Radix rubiginosa*	Yes (1–4%)	— (matched to same species GU451737 at 95%)	Yes (1–4%)	*Radix rubiginosa* U82076.2 (100)
Nassariidae	*Anentome bockii*	Yes (1–4%)	—	N/A	N/A
	*Anentome helena*	Yes (1–4%)	—	N/A	N/A
Neritidae	*Clithon corona*	Yes (1–3%), no (4%)	— (matched to same species EU732362 at 93%)	N/A	N/A
	*Clithon diadema*	Yes (1–4%)	—	Yes (1–3%), no (4%)	*Clithon spinosus* AY771224 (96)
	*Clithon lentiginosum*	Yes (1–3%), no (4%)	—	Yes (1–4%)	—
	*Clithon mertoniana*	Yes (1–4%)	—	Yes (1–3%), no (4%)	—
	*Neripteron auriculata*	Yes (1–4%)	—	Yes (1–4%)	—
	*Neritina iris*	Yes (1–4%)	—	N/A	N/A
	*Neritina juttingae*	Yes (1–4%)	—	Yes (1–4%)	—
	*Neritina violacea*	Yes (1–4%)	*Neritina violacea* JX411691 (99)	N/A	N/A
	*Neritodryas cornea*	Yes (1–4%)	—	N/A	N/A
	*Septaria porcellana*	N/A	N/A	Yes (1–4%)	*Septaria porcellana* AY771228 (98)
	*Vittina coromandeliana*	Yes (1–4%)	*Neritina turrita* JX411698 (99)	N/A	N/A
	*Vittina turrita*	Yes (1–4%)	—	N/A	N/A
	*Vittina waigiensis*	Yes (1–4%)	*Neritina turrita* AY820497 (100)	Yes (1–4%)	—
Pachychilidae	*Brotia armata*	N/A	N/A	No (1–4%)	*Brotia armata* AY330810 (100)
	*Brotia binodosa*	N/A	N/A	No (1–4%)	*Brotia* sp. FJ377079 (100)
	*Brotia herculea*	Yes (1–4%)	—	N/A	N/A
	*Sulcospira tonkiniana*	Yes (1–4%)	*Sulcospira tonkiniana* FJ377297 (99)	N/A	N/A
	*Tylomelania towutica*	Yes (1–4%)	*Tylomelania towutica* KJ850891 (99)	N/A	N/A
	*Tylomelania* sp.	Yes (1–4%)	*Tylomelania* sp. KJ850861 (99)	Yes (1–4%)	*Tylomelania centaurus* AY311829 (99)
Physidae	*Physa* sp.	Yes (1–4%)	—	N/A	N/A
Planorbidae	*Gyraulus convexiusculus*	Yes (1–4%)	—	N/A	N/A
	*Indoplanorbis exustus*	Yes (1–4%)	*Indoplanorbis exustus* GU451743 (99)	N/A	N/A
Semisulcospiridae	*Semisulcospira* sp.	Yes (1–4%)	—	N/A	N/A
Thiaridae	*Melanoides tuberculata*	Yes (1–4%)	*Melanoides tuberculata* KP284138 (100)	N/A	N/A
	*Stenomelania offachiensis*	Yes (1–4%)	—	Yes (1–4%)	*Stenomelania* sp. AY010518 (97)
	*Stenomelania plicaria*	Yes (1–4%)	—	N/A	N/A
	*Stenomelania* cf. *plicaria*	Yes (1–4%)	—	N/A	N/A
	*Stenomelania* sp.	Yes (1–4%)	—	Yes (1–4%)	—
	*Thiara cancellata*	Yes (1–4%)	—	Yes (1–4%)	—
Viviparidae	*Filopaludina cambodjensis*	Yes (1–4%)	—	N/A	N/A
	*Filopaludina peninsularis*	Yes (1–4%)	—	Yes (1–4%)	—
	*Sinotaia guangdungensis*	Yes (1–4%)	*Bellamya heudei guangdungensis AY296827 (99)	N/A	N/A
	*Taia pseudoshanensis*	N/A	—	Yes (1–4%)	—

“N/A” indicates barcodes were not obtained; “—”indicate that top hits on BLAST were <96%.

*Bellamya heudei guangdungensis is a synonym of *Sinotaia guangdugensis* [73].

For matches to GenBank, only 17 species had 97–100% identity matches to available CO1 sequences in GenBank (Table 4), and 13 of those had species names that matched those assigned based on morphology. For the other four species that did not match the morphology-based identities—*Corbicula moltkiana* and *Parreysia tavoyensis* were only identified to genus level based on morphology, and were confirmed based on the GenBank hits [61, 84], with additional comparison to the original descriptions; two individuals identified by morphology as *Vittina coromandeliana* and *Vittina waigiensis* were 99–100% matched to two separate submissions on GenBank that were identified as *Vittina turrita*. Neither study included photographs of the species, nor could the sequenced individuals be located ([85], [86], M.B. Goodwin, pers. comm.); because the two GenBank sequences for *Vittina turrita* were separated by a 4.5% uncorrected pairwise distance, we retained our morphology-based identifications for *Vittina coromandeliana* and *Vittina waigiensis*. Among the 27 CO1 sequences that fell outside the 3% species delimitation threshold, three had top hits with species that matched the morphology-based identification, but were only 90–95% matches—*Pilsbryoconcha exilis*, *Radix rubiginosa*, and *Clithon corona* (Table 4). For the other 24 sequences, the top hits had between 78 and 94% identity matches, and all the top hits were for the same family as the original (morphology-based) identification (S2 Table). For 16S, 10 species had 97–100% identity matches to sequences on GenBank (Table 4)—five of which exactly matched the species identified based on morphology, while the other five were matched to congeners. For the remaining 14 sequences, the top hits had between 86 and 96% identity matches, and all were of the same family as the original identification.

## Discussion

This study represents the first comprehensive survey of freshwater mollusc species in the ornamental pet trade. We found an unexpectedly high diversity of species in the trade over the six-year period, which suggests an increase in interest in ornamental freshwater molluscs, mirroring the increased demand for invertebrates elsewhere [5, 7]. Our results highlight the role of the ornamental pet trade as a key anthropogenic factor influencing both introductions (of non-native molluscs, and associated parasites), and the conservation of freshwater molluscs.

### Invasion potential

The aquarium or ornamental pet trade has been suspected to be the main pathway for freshwater alien species introductions into Singapore [45, 87]. The results of the present survey indicate that nearly half of known introduced and species of unknown origin in Singapore are in the trade [47, 48,72, 73, 88]. More importantly, the study found evidence for the indirect introduction of species via other ornamental taxa [89, 90]—more than half of the species recorded as hitchikers in the trade are established in Singapore. Singapore is also a hub for the import and export of ornamental aquatic plants, and freshwater snails have been detected among shipments of plants from the country [19, 43, 44, 90]. It is likely that even species that are not attractive to hobbyists (e.g., species <10 mm in size) could still be imported, and subsequently introduced into the environment through indiscriminate disposal of aquarium water [14], or planting of ornamental plants around waterbodies.

Among the 14 species with a history of introduction elsewhere, 12 were purportedly introduced via the ornamental pet trade (Table 3). The two species with no prior records of introduction through the trade, *Sinanodonta woodiana* and *Sinotaia guangdungensis*, were likely co-introduced with fish [47, 73, 91, 92]. The ornamental species with recorded or potential impacts include some of the world’s most notorious invasive species—*Corbicula fluminea* and *Pomacea canaliculata* (likely *Pomacea maculata* too) [93, 94]. These species have caused environmental as well as economic damage throughout their introduced ranges, including habitat modification, agricultural losses, and possible displacement of native species (Table 3). Recognising the threat of *Pomacea* spp., commonly known as golden apple snails, the European Commission recently banned their import into the European Union [95]. Our results show that the authorities may need to expand their monitoring efforts to include other similarly invasive species like *Sinanodonta woodiana* and *Corbicula fluminea*.

Additionally, the spread of snail-mediated zoonotic diseases such as schistosomiasis with the introduction of freshwater molluscs via the ornamental pet trade has been a cause for concern for many decades [19, 96, 97], and our results highlight that the threat remains—half of the species with introduction impacts are also known intermediate hosts of parasites that can cause diseases in humans and livestock, with disease outbreaks impacting humans or fisheries being recorded following the introduction of *Pomacea* spp. and *Melanoides tuberculata* (Table 3). Besides the species with introduction history that are known intermediate hosts of zoonotic parasites, snails of the family Bithyniidae, of which at least one species is in the trade, are also known parasite vectors [98].

### Origins of molluscs in the trade and conservation concerns

Of the 59 species recorded, more than half are naturally distributed in the Oriental region, while almost a fifth have a more widespread distribution that includes the Oriental region. This could be because the Oriental region has the highest diversity for snails of the families Neritidae, Thiaridae, Pachychilidae, and Viviparidae [24], which made up almost 90% of the recorded ornamental mollusc species. The Oriental region is also one of only two global hotspots of Unionidae bivalve diversity and endemism (the other being the southeastern United States) [23]. The higher proportion of species from the Oriental region contrasts with the freshwater fishes in the Singapore ornamental pet trade, which mostly originate from the Neotropics [99].

The high diversity of freshwater molluscs in the trade originating from the Oriental region may be of conservation concern, especially considering the freshwater molluscs in the region are understudied—with taxonomic status and distributions of many species being uncertain [25]. Fewer than half of the species in the ornamental pet trade (22 Oriental species and two Neotropical species) have been assessed by the IUCN. Three species are listed as Data Deficient, while many of the 21 Least Concern species lack updated population information [83]. An exception among the Least Concern species is *Hyriopsis bialata*, which has a decreasing population trend in Thailand [100]. Although population trends are unknown for the other Least Concern species, there have been accounts of localized threats, for e.g., degradation of *Corbicula moltkiana* habitat in Indonesia, and multiple threats of habitat loss, pollution, and overharvesting potentially impacting the Vietnamese populations of *Pilsbryoconcha exilis* [83].

Overharvesting for human consumption is a known threat to freshwater molluscs in the Oriental region, especially for narrowly endemic species [25, 83]. Although harvesting for the ornamental pet trade has thus far not been documented to be a threat, we recorded seven out of nine pachychilid and one viviparid species in the trade with restricted ranges; specifically, *Brotia armata*, *Brotia binodosa*, *Brotia pagodula* which are confined to particular river systems in Indo-Burma [63, 101], and all *Tylomelania* spp. and *Celetaia persculpta*, which are endemic to Lake Poso of Sulawesi ([102], T. von Rintelen pers. comm.). These species appeared to fetch higher prices compared to more common species (up to US$10 per individual *Tylomelania* sp. compared to <US$5 per individual Thiaridae or Neritidae, THN pers. obs.). The rarity of the species may drive increased demand, which may ultimately lead to a decline of the species [103].

Besides the huge knowledge gaps in population trends, taxonomic uncertainty is also a factor that may mask the true distribution of species [104]. *Clithon corona* is one of the species in the trade that has been assessed as Least Concern and is assumed to be widely distributed; however, its taxonomy, and thus its true distribution, remains unresolved [75, 83]. The limited knowledge of species distributions, restricted ranges, and unresolved taxonomy of freshwater molluscs in the ornamental pet trade makes it essential that the trade be monitored more closely lest the harvesting of species for the trade becomes unsustainable.

### Utility of DNA barcodes

Introduction pathways are difficult to track; many introductions fail to be detected because in many cases, the appropriate expertise is not available for identifying molluscs to species. While it is currently not feasible to rely wholly on molecular methods for species identification and delimitation [105] (see below regarding limitations), the fact remains that there is a global decline in trained taxonomists, and funding for necessary resources and educational support [106]. This lack of expertise affects all taxa, including molluscs [25, 26], and is especially crucial in relation to the ornamental pet trade. Border security officials are given the insurmountable task to inspect large volumes of often poorly-categorised or wrongly identified shipments [13, 107].

It is here that DNA barcodes provide useful information. In general, identification of species via DNA barcodes can be viewed as a two-stop process. First, the samples can be delimited into mOTUs via genetic distance and it is generally found that most mOTUs are stable across a large range of distances. Next, the mOTUs are identified to species either by matching sequences to an existing database or by expert morphological examination. It is at this stage that conflict between data sources can be detected and often resolved. Our results support the use of DNA barcoding for rapid species delimitation, especially for species in the ornamental pet trade, which originate from multiple (and often unknown) sources. However, we acknowledge our DNA database is currently still too incomplete for routine identification of many species that lack coverage. This has been a problem for many taxa, and researchers around the world are tackling this issue via rapid generation of name-matched DNA barcodes [108, 109].

**Fig 3:**
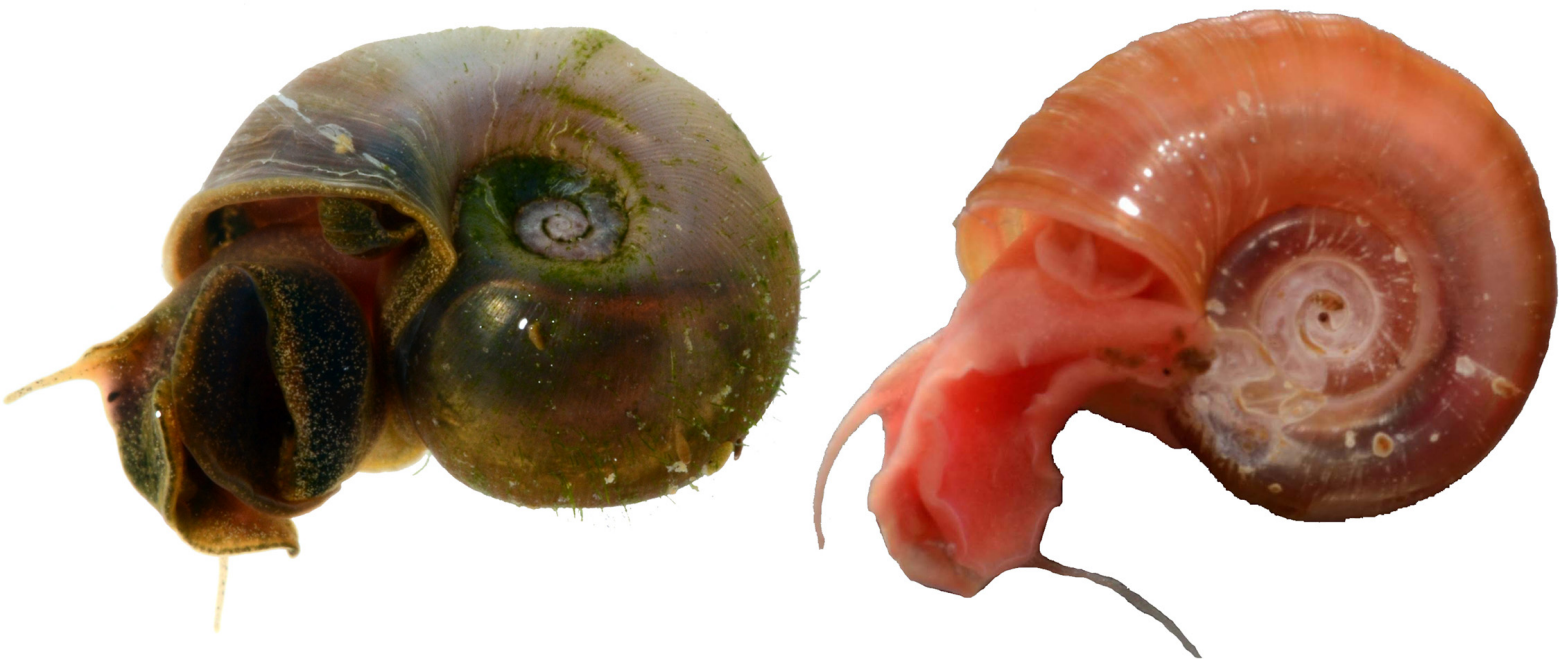
Two morphs of *Indoplanorbis exustus* in the aquarium trade.

The process of amplifying and extracting DNA from freshwater molluscs is often difficult owing to the presence of additional mucopolysaccharides in the slime that inhibits PCR [110]. However, we show that even with short DNA fragments (≤ 313 bp), species can be reliably matched to existing sequences on GenBank, e.g., *Melanoides tuberculata* (CO1, 239 bp), or distinguished from congeners, e.g., *Vittina turrita* and *Vittina waigiensis* which were separated by 10% genetic divergence (CO1, both 313 bp). DNA barcodes also allowed for more accurate identification of some species (e.g., confirmation of identification for *Corbicula moltkiana* and *Parreysia tavoyensis*). In addition, DNA barcodes are usually less variable than the morphology of wild and cultured individuals, which makes rapid identification based solely on external morphology difficult and sometimes unreliable. For example, two morphologically distinct forms of *Indoplanorbis* sp. were collected from the trade (Fig 3), but shared identical DNA barcodes (GenBank Accession numbers KU318341–42), and were instead then considered conspecific (*Indoplanorbis exustus*).

DNA barcodes work well when the chosen molecular markers are sufficiently diagnostic to delimit species [111] and the species taxonomy of species is well resolved [111]; most species in our study satisfied these conditions. Some exceptions, however, were closely-related, sympatric species (*Tylomelania* spp., *Brotia armata*, *Brotia binodosa*) that failed to have diagnostic barcodes, possibly due to either recent speciation or to introgression [63]. Our study also included species with poorly understood species boundaries; a good example is in the case of three *Stenomelania* spp. in the trade which belong to the *Stenomelania plicaria* species complex ([112], M. Glaubrecht pers. comm.). DNA barcodes can here suggest species boundaries, but they have to be confirmed via taxonomic revision. Completing this task will be particularly important for some invasive molluscs with high genetic variability [113]. Another problem with DNA barcodes are misidentified sequences in barcode databases [114–117], which was encountered here for two *Vittina* species that matched two separate sequences identified as *Vittina turrita* on GenBank. The lack of supporting voucher specimens or photographs made it impossible to resolve the conflict and is characteristic of many misidentifications in barcode databases.

Because of these known problems, we made sure to identify specimens using both morphology and DNA sequences [118], and deposit voucher specimens in the Lee Kong Chian Natural History Museum for future validation or reconfirmation. In any case, despite the discussed limitations in the utility of mitochondrial DNA, it presently remains the most suitable marker for rapid identification, especially in the case of monitoring the ornamental pet trade [33, 34]. It is hoped that this study would serve as a start for building a more complete DNA barcode library for freshwater molluscs in the ornamental pet trade.

### Conclusions

We believe that our assessment is representative of freshwater molluscs that are currently in the trade, but the ornamental pet trade changes over time [6, 7] and it would be important to monitor the trade on a regular basis. It would be prudent to continue building up a reliable DNA barcode library, as barcodes would be extremely useful to rapidly identify species in limited availability or absence of taxonomic expertise. Future work should take advantage of new amplification techniques [119], and cheap NGS barcodes obtained with next-generation sequencing [109], which lower the cost for obtaining barcodes, and will allow for the inclusion of population genomics for tracing the origin and spread of species that have been widely introduced. Also, as indirect introductions via the importation of aquatic plants appear to be overlooked, this pathway warrants more in-depth attention. In light of the recent European Union regulations against *Pomacea* spp., it would be in the interest of major ornamental distributors, such as Singapore, to prevent the export of unwanted molluscs with aquatic plants. Current knowledge gaps in the autecology of freshwater molluscs (especially those in the Oriental region) need to be filled to identify high invasive risk species that are commonly translocated via this pathway. For example, it would be important to know how long mollusc eggs remain viable on plants. Besides helping the industry to prevent import and monitor or manage the spread of potentially invasive species, such ecological information would also help inform management and conservation of endemic freshwater molluscs, thereby creating a less harmful and more sustainable ornamental pet trade.

## Supporting Information

S1 Table Sources of ornamental freshwater molluscs (local ornamental pet retail shops and major ornamental exporters) (DOCX)

S2 Table GenBank and BOLD Accession Numbers for COI and 16S sequences of freshwater molluscs of the ornamental pet trade. (DOCX)
