# Supplementary material for: Molluscs for Sale: Assessment of Freshwater Gastropods and Bivalves in the Ornamental Pet Trade
Source: PLoS One. 2016 Aug 15;11(8):e0161130. doi: 10.1371/journal.pone.0161130 (PMC4985174; doi:10.1371/journal.pone.0161130)
Supplement: S1 Table — (DOCX) [file pone.0161130.s001.docx]

**S1 Table. Sources of ornamental freshwater molluscs (local ornamental pet retail shops and major ornamental exporters)**

| **Name of Company** | **Address** |
| --- | --- |
| Aquaculture Technologies Pte. Ltd. | 2 Kim Chuan Drive, Singapore 537080 |
| Clementi Florist and Aquarium | Block 328, Clementi Avenue 2, #01–210, Singapore 120328 |
| Nanyang Trading Aquarium Pte. Ltd. | 2 Seletar West Farmway, Singapore 798098 |
| Polyart Aquarium | Block 328, Clementi Avenue 2, #01–194, Singapore 120328 |
| Qian Hu Fish Farm Trading | 71, Jalan Lekar, Sungei Tengah, Singapore 698950 |
| Sunbeam Aquarium Pte. Ltd. | 9 Chencharu Link, Singapore 768147 |
| Wu Hu Aquarium | 71 Seng Poh Road, #01-39, Singapore 160071 |
