# Supplementary material for: Molluscs for Sale: Assessment of Freshwater Gastropods and Bivalves in the Ornamental Pet Trade
Source: PLoS One. 2016 Aug 15;11(8):e0161130. doi: 10.1371/journal.pone.0161130 (PMC4985174; doi:10.1371/journal.pone.0161130)
Supplement: S2 Table — (DOCX) [file pone.0161130.s002.docx]

**S2 Table. GenBank and BOLD Accession Numbers for COI and 16S sequences of freshwater molluscs of the ornamental pet trade.**

| **Family** | **Species** | **BOLD Process ID** | **CO1** | | | | | **16S** | | | | |
| --- | --- | --- | --- | --- | --- | --- | --- | --- | --- | --- | --- | --- |
|  |  |  | **GenBank Accession No.** | **Family** | **Species of the highest-score hits** | **Identity (%)** | **Coverage (%)** | **GenBank Accession No.** | **Family** | **Species of the highest-score hits** | **Identity (%)** | **Coverage (%)** |
| Cyrenidae | *Batissa violacea* | AQMOL001-16 | KU318372 | Cyrenidae | *Batissa violacea* DQ837727 | 97 | 92 | KU318348 | Cyrenidae | *Corbicula japonica* AB304507 | 86 | 100 |
|  | *Corbicula* cf. *fluminea* | AQMOL002-16 | KU318325 | Cyrenidae | *Corbicula* sp. GU781082 | 100 | 99 | KU318349 | Cyrenidae | *Corbicula colorata* JX399588 | 99 | 100 |
|  | *Corbicula moltkiana* | AQMOL003-16 | KU318373 | Cyrenidae | *Corbicula moltkiana* AY275660.1 | 100 | 93 | KU318350 | Cyrenidae | *Corbicula largillierti* AB522658 | 99 | 100 |
| Unionidae | *Hyriopsis bialata* | AQMOL004-16 | KU318374 | Unionidae | *Contradens contradens* DQ191411 | 88 | 86 | — | — | — | — | — |
|  | *Hyriopsis desowitzi* | AQMOL046-16 | — | — | — | — | — | KU318351 | Unionidae | *Contradens contradens* KP795054 | 86 | 100 |
|  | *Parreysia burmana* | AQMOL005-16 | KU318375 | Unionidae | *Radiatula khadakvaslaensis* KF690124 | 89 | 93 | KU318352 | Unionidae | *Parreysia olivacea* KP795044 | 96 | 98 |
|  | *Parreysia tavoyensis* | AQMOL006-16 | KU318376 | Unionidae | *Parreysia tavoyensis* JN243901 | 99 | 76 | KU318353 | Unionidae | *Parreysia tavoyensis* KP795043 | 99 | 94 |
|  | *Pilsbryoconcha exilis* | AQMOL007-16 | KU318326 | Unionidae | *Pilsbryoconcha exilis* KP795024 | 90 | 91 | — | — | — | — | — |
|  | *Scabies crispata* | AQMOL047-16 | — | — | — | — | — | KU318354 | Unionidae | *Scabies crispata* KP795048 | 97 | 100 |
|  | *Sinanodonta woodiana* | AQMOL008-16 | KU318377 | Unionidae | *Sinanodonta woodiana* KJ434487 | 99 | 88 | — | — | — | — | — |
| Ampullariidae | *Marisa cornuarietis* | AQMOL009-16 | KU318378 | Ampullariidae | *Marisa cornuarietis* KM100140 | 98 | 91 | — | — | — | — | — |
|  | *Pomacea canaliculata* | AQMOL010-16 | KU318379 | Ampullariidae | *Pomacea canaliculata* KJ739609 | 99 | 99 | — | — | — | — | — |
|  | *Pomacea diffusa* | AQMOL011-16 | KU318327 | Ampullariidae | *Pomacea diffusa* EF515065 | 100 | 96 | — | — | — | — | — |
| Bithyniidae | *Bithynia* sp. | AQMOL012-16 | KU318328 | Pachychilidae | *Brotia peninsularis* AY330851 | 87 | 93 | KU318355 | Bithynidae | *Bithynia tentaculata* JX970531 | 88 | 100 |
| Lymnaeidae | *Radix rubiginosa* | AQMOL015-16 | KU318330 | Lymnaeidae | *Radix rubiginosa* GU451737 | 95 | 98 | KU318356 | Lymnaeidae | *Radix rubiginosa* U82076.2 | 100 | 100 |
| Nassariidae | *Anentome bockii* | AQMOL013-16 | KU318380 | Nassariidae | *Nassaria* sp. GU439989 | 87 | 97 | — | — | — | — | — |
|  | *Anentome helena* | AQMOL014-16 | KU318329 | Nassariidae | *Nassarius siquijorensis* EU124793 | 87 | 100 | — | — | — | — | — |
| Neritidae | *Clithon corona* | AQMOL017-16 | KU318331 | Neritidae | *Clithon corona* EU732362 | 93 | 95 | — | Neritidae | *Clithon spinosus* AY771224 | 96 | 95 |
|  | *Clithon diadema* | AQMOL018-16 | KU318332 | Neritidae | *Clithon spinosus* AF236070 | 94 | 93 | KU318357 | Neritidae | *Clithon spinosus* AY771224 | 94 | 97 |
|  | *Clithon lentiginosum* | AQMOL019-16 | KU318333 | Neritidae | *Clithon corona* EU732362 | 94 | 96 | KU318358 | Neritidae | *Clithon spinosus* AY771224 | 95 | 100 |
|  | *Clithon mertoniana* | AQMOL020-16 | KU318334 | Neritidae | *Clithon corona* EU732362 | 90 | 94 | KU318359 | Neritidae | *Septaria sanguisuga* AY771229 | 93 | 100 |
|  | *Neripteron auriculata* | AQMOL021-16 | KU318381 | Neritidae | *Neritina violacea* JX411681 | 93 | 95 | KU318360 | — | — | — | — |
|  | *Neritina iris* | AQMOL022-16 | KU318382 | Neritidae | *Neritina canalis* GU001366 | 93 | 99 | — | Neritidae | *Theodoxus prevostianus* AY771254 | 93 | 97 |
|  | *Neritina juttingae* | AQMOL023-16 | KU318383 | Neritidae | *Neritina violacea* DQ060926 | 88 | 100 | KU318361 | — | — | — | — |
|  | *Neritina violacea* | AQMOL024-16 | KU318335 | Neritidae | *Neritina violacea* JX411691 | 99 | 93 | — | — | — | — | — |
|  | *Neritodryas cornea* | AQMOL048-16 | KU318384 | Neritidae | *Nerita albicilla* EU253393 | 86 | 99 | — | Neritidae | *Septaria porcellana* AY771228 | 98 | 100 |
|  | *Septaria porcellana* | AQMOL025-16 | — | — | — | — | — | KU318362 | — | — | — | — |
|  | *Vittina coromandeliana* | AQMOL026-16 | KU318336 | Neritidae | *Neritina turrita* JX411698 | 99 | 91 | — | — | — | — | — |
|  | *Vittina turrita* | AQMOL027-16 | KU318385 | Neritidae | *Vittina variegata* EU732371 | 91 | 96 | — | Neritidae | *Neritina virginea* JX646659 | 95 | 100 |
|  | *Vittina waigiensis* | AQMOL049-16 | KU318386 | Neritidae | *Neritina turrita* AY820497 | 100 | 89 | KU318363 | Pachychilidae | *Brotia armata* AY330810 | 100 | 100 |
| Pachychilidae | *Brotia armata* | AQMOL050-16 | — | — | — | — | — | KU318364 | Pachychilidae | *Brotia* sp. FJ377079 | 100 | 100 |
|  | *Brotia binodosa* | AQMOL028-16 | — | — | — | — | — | KU318365 | — | — | — | — |
|  | *Brotia* aff. *herculea* | AQMOL029-16 | KU318347 | Pachychilidae | *Brotia dautzenbergiana* AY330831 | 90 | 92 | — | — | — | — | — |
|  | *Sulcospira tonkiniana* | AQMOL031-16 | KU318337 | Pachychilidae | *Sulcospira tonkiniana* FJ377297 | 99 | 97 | — | — | — | — | — |
|  | *Tylomelania towutica* | AQMOL032-16 | KU318338 | Pachychilidae | *Tylomelania towutica* KJ850891 | 99 | 97 | — | — | — | — | — |
|  | *Tylomelania* sp. | AQMOL033-16 | KU318339 | Pachychilidae | *Tylomelania* sp. KJ850861 | 99 | 98 | KU318366 | Pachychilidae | *Tylomelania centaurus* AY311829 | 99 | 90 |
| Physidae | *Physa* sp. | AQMOL034-16 | KP182981 | Physidae | *Physella acuta* KF966541 | 78 | 100 | — | — | — | — | — |
| Planorbidae | *Gyraulus convexiusculus* | AQMOL035-16 | KU318340 | Planorbidae | *Gyraulus* sp. KC495833 | 93 | 94 | — | — | — | — | — |
|  | *Indoplanorbis exustus* | AQMOL036-16 | KU318342 | Planorbidae | *Indoplanorbis exustus* GU451743 | 99 | 98 | — | — | — | — | — |
| Semisulcospiridae | *Semisulcospira* sp. | AQMOL037-16 | KU318389 | Thiaridae | *Paludomus siamensis* AY456560 | 91 | 99 | — | — | — | — | — |
| Thiaridae | *Melanoides tuberculata* | AQMOL038-16 | KU318388 | Thiaridae | *Melanoides tuberculata* KP284138 | 100 | 100 | — | Thiaridae | *Stenomelania* sp. AY010518 | 97 | 92 |
|  | *Stenomelania offachiensis* | AQMOL039-16 | KU318343 | Thiaridae | *Melanoides admirabilis* AY456561 | 90 | 95 | KU318367 | — | — | — | — |
|  | *Stenomelania plicaria* | AQMOL040-16 | KU318344 | Thiaridae | *Melanoides tuberculata* AY575974 | 89 | 93 | — | — | — | — | — |
|  | *Stenomelania* cf. *plicaria* | AQMOL041-16 | KU318390 | Thiaridae | *Stenomelania rufescens* EU273767 | 92 | 100 | — | Thiaridae | *Thiara amarula* AY010520 | 93 | 100 |
|  | *Stenomelania* sp. | AQMOL042-16 | KU318391 | Thiaridae | *Stenomelania uniformis* EU273762 | 94 | 98 | KU318368 | Thiaridae | *Thiara amarula* AY010520 | 94 | 100 |
|  | *Thiara cancellata* | AQMOL043-16 | KU318345 | Thiaridae | *Thiara scabra* KP774719 | 85 | 96 | KU318369 | — | — | — | — |
| Viviparidae | *Filopaludina cambodjensis* | AQMOL044-16 | KU318392 | Viviparidae | *Bellamya bengalensis* FJ405877 | 91 | 92 | — | Viviparidae | *Bellamya bengalensis* FJ405735 | 90 | 93 |
|  | *Filopaludina peninsularis* | AQMOL045-16 | KU318346 | Viviparidae | *Bellamya bengalensis* FJ405877 | 90 | 87 | KU318370 | — | — | — | — |
|  | *Sinotaia guangdungensis* | AQMOL051-16 | KU318393 | Viviparidae | *Bellamya heudei guangdungensis* AY296827 | 99 | 97 | — | Viviparidae | *Bellamya* cf. *capillata* JX489318 | 88 | 100 |
|  | *Taia pseudoshanensis* | AQMOL001-16 | — | — | — | — | — | KU318371 |  |  |  |  |
